# Epicardial cavernous haemangioma; A case report of a unique incidental finding

**DOI:** 10.1093/ehjcr/ytae146

**Published:** 2024-03-21

**Authors:** Rayan Cheaban, Misagh Piran, Dragan Opacic, Jan F Gummert, Sebastian V Rojas

**Affiliations:** Clinic for Thoracic and Cardiovascular Surgery, Herz- und Diabeteszentrum Nordrhein-Westfalen, Universitätsklinik der Ruhr-Universität Bochum, Med. Fakultät OWL (Universität Bielefeld), Georgstraße 11, 32545 Bad Oeynhausen, Germany; Clinic for Radiology, Nuclear Medicine and Molecular Imaging, Heart and Diabetes Center North Rhine Westphalia, University Hospital, Ruhr-University Bochum, Bad Oeynhausen, Germany; Clinic for Thoracic and Cardiovascular Surgery, Herz- und Diabeteszentrum Nordrhein-Westfalen, Universitätsklinik der Ruhr-Universität Bochum, Med. Fakultät OWL (Universität Bielefeld), Georgstraße 11, 32545 Bad Oeynhausen, Germany; Clinic for Thoracic and Cardiovascular Surgery, Herz- und Diabeteszentrum Nordrhein-Westfalen, Universitätsklinik der Ruhr-Universität Bochum, Med. Fakultät OWL (Universität Bielefeld), Georgstraße 11, 32545 Bad Oeynhausen, Germany; Clinic for Thoracic and Cardiovascular Surgery, Herz- und Diabeteszentrum Nordrhein-Westfalen, Universitätsklinik der Ruhr-Universität Bochum, Med. Fakultät OWL (Universität Bielefeld), Georgstraße 11, 32545 Bad Oeynhausen, Germany

**Keywords:** Primary cardiac tumour, Haemangioma, Incidentally diagnosed cardiac mass, Multimodality imaging, Surgical treatment, Case report

## Abstract

**Background:**

Primary cardiac tumours are rare, accounting for only 0.002–0.03% at autopsy. Cardiac haemangiomas are benign vascular tumours and constitute for 0.28% of all primary cardiac tumours. Cavernous haemangiomas, capillary haemangiomas, and arteriovenous haemangiomas are three distinct types. Cardiac haemangiomas are often misdiagnosed as myxomas and must be differentiated from malignant angiosarcomas.

**Case summary:**

We present a 44-year-old Mediterranean male patient with a cavernous haemangioma in the inferior vena cava and right atrium, detected on transthoracic echocardiography. The patient experienced palpitations and dyspnoea on exertion. Computed tomography (CT) angiography revealed a 7.5 × 6 × 5 cm mass suspected to be perfused by the distal right coronary artery. A watch-and-wait approach was suggested, leading to a cardiac magnetic resonance imaging (MRI) with contrast 6 months later. T_1_ mapping exhibited a prolonged relaxation time and isointensity to the myocardium. T_2_ mapping revealed a homogenous hyperintense mass with heterogenous late enhancement. Surgical excision was performed using a bicaval cannulation technique on cardiopulmonary bypass. Intraoperatively, no connection to the coronaries was noted. At 1 year follow-up, the patient reported restored physical resilience, with no evidence of tumour recurrence.

**Discussion:**

Clinical symptoms of cardiac cavernous haemangiomas are unspecific and become evident once the tumour grows. To investigate the nature and vascular involvement of the tumour, a contrast-enhanced CT angiography or MRI can be performed. Cardiac haemangiomas are often misdiagnosed and must be differentiated from malignant angiosarcomas. Clear guidelines for the treatment of cardiac haemangiomas in adult patients are lacking. Primary cardiac tumours require thorough investigation, and surgical intervention should be tailored to the individual’s case.

**Learning points**

- Emphasizing the significance in differentiating cardiac masses and understanding its nature pre-operatively is crucial, utilizing CT, MRI, and coronary catheterization.
- Although haemangiomas are usually benign, their growth can give rise to life-threatening complications, underlining the importance of proactive management.
- Surgical excision stands as a recommended primary approach in adult patients due to its favourable outcomes and low recurrence rates, supported by multiple case reports.

## Introduction

Primary cardiac tumours are scarce, accounting for 0.002–0.03% cases at autopsy,^1^ with myxoma being the most common type.^2^ Cardiac haemangiomas are typically diagnosed between the age of 40 and 49 years old^3^; however, it may occur at any age.^1^ As primary cardiac tumours, haemangiomas are rarely seen (2.8% of all cardiac tumours).^3-5^ While haemangiomas are commonly associated with the skin, they can also affect internal organs.^6^ They are classified into three types: cavernous, capillary, and arteriovenous haemangiomas. Histologically, this benign structure is characterized by large, dilated blood vessel proliferation lined by flat endothelial cells.^3,7^ Li *et al.*^3^ reviewed 200 case reports about patients with cardiac haemangiomas, revealing that 26.2% of all cardiac haemangiomas occur in the right atrium and 23.1% in the left ventricle. They are rarely found in the epicardium.^8,9^ These tumours present with unspecific clinical symptoms that become evident as the tumour grows,^10^ potentially leading to obstructive symptoms and life-threatening complications such as invasion of the cardiac conduction system, cardiac tamponade, and even sudden death.^1,6,10^ Cardiac haemangiomas are often misdiagnosed as myxomas and must be differentiated from malignant angiosarcomas.^1,11^

## Summary figure

**Figure:**
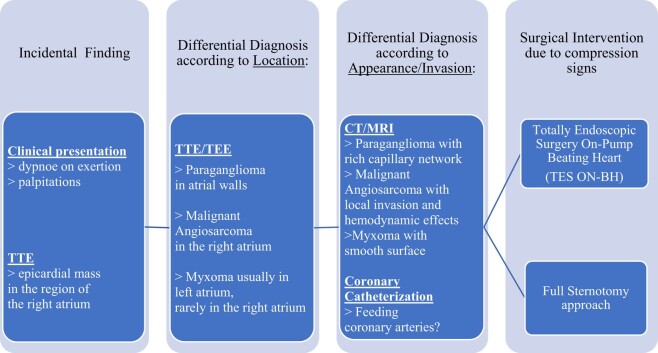


## Case summary

**Figure 1:**
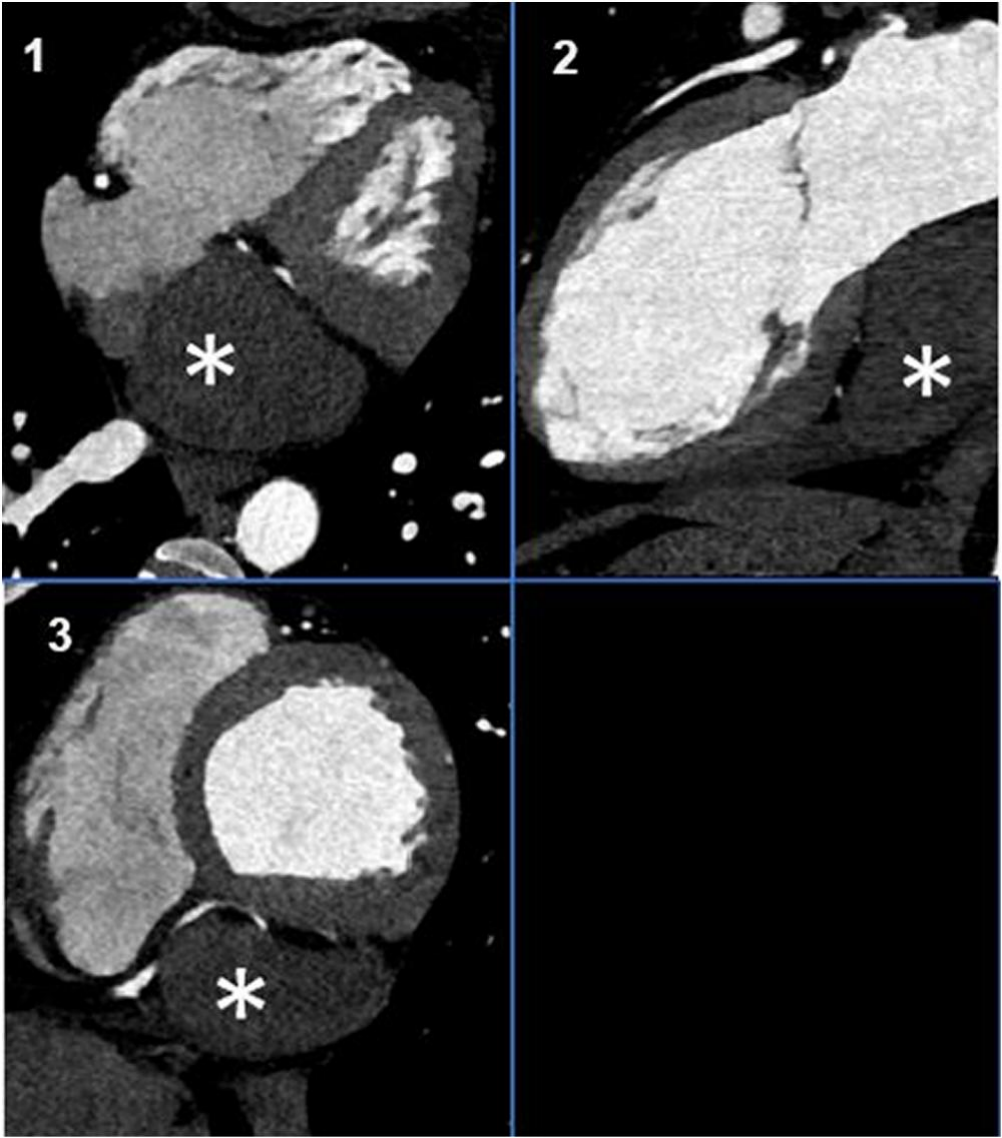
Contrast-enhanced computed tomography angiography of a cardiac cavernous haemangioma measuring 7.5 × 6 × 5 cm (*): The cavernous haemangioma is non-enhancing and appears as a hypodense heterogenous mass presented in different planes. (1) Four-chamber view. (2) Long-axis two-chamber view. (3) Short-axis view.

We are presenting a 44-year-old Mediterranean male patient who was admitted to our clinic presenting an epicardial tumour in the region of the inferior vena cava extending to the right atrium and a coronary sinus. He had no family history of cancer or cardiovascular diseases, no cardiovascular risk profile, or history of co-morbid illnesses. Nine months prior to surgery, he experienced palpitations and dyspnoea on exertion, classified as NYHA Class II. On auscultation, no pathological heart sounds emerged. A 12-lead ECG showed a normal sinus rhythm with a heart rate of 62 b.p.m., with unremarkable PR and QRS segments. No skin lesions were observed. The tumour was incidentally detected during a transthoracic echocardiography (TTE), disclosing a hyperechoic epicardial structure measuring 6 × 4 cm (see Supplementary material online, *Video 1*). A contrast-enhanced computed tomography (CT) angiography revealed a hypodense heterogenous mass measuring 7.5 × 6 × 5 cm (*Figure 1*). It was suggested to be a paraganglionic mass that was vascularized by the distal right coronary artery (RCA), which could not be ruled out during coronary catheterization. A watch-and-wait approach was suggested to monitor any progression of the tumour.

**Figure 2:**
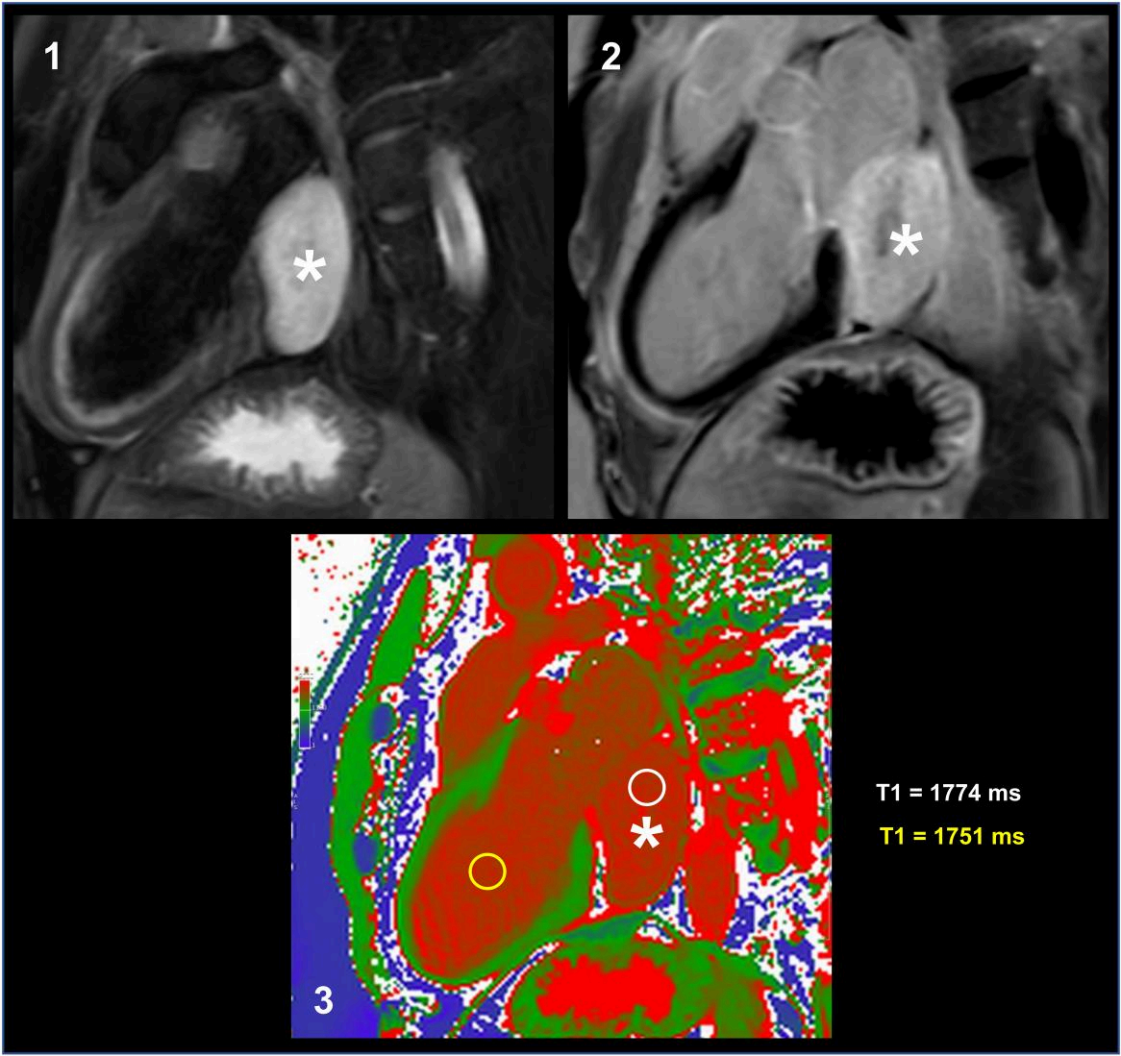
Magnetic resonance tomography of the cardiac cavernous haemangioma (*): (1) T_2_-weighted image: A haemangioma is a tumour of blood vessel proliferation. Blood has a long relaxation time. Thus, the tumour appears in a T_2_-weighted image as a homogenous hyperintense mass. (2) Late gadolinium enhancement using phase-sensitive inversion recovery: Healthy myocardium null or suppress the blood signal in late enhancement phase (black). The myocardium has a different relaxation time than the tumour. In late gadolinium enhancement the tumour appears as a hyperintense mass. (3) T_1_ colour mapping: Native myocardium has a field strength of 3 T and a T_1_ relaxation time of ∼1240 ms (green). Left ventricular blood has a T_1_ relaxation time of 1774 ms (red). The tumour has a similar T_1_ relaxation time of 1751 ms (red) as the left ventricular blood. We used T_1_ colour mapping to estimate the relaxation time after contrast enhancement. For T_2_ phase, we did not estimate the relaxation time, and thus, T_2_ colour mapping was not performed.

Six months later, a magnetic resonance imaging (MRI) with contrast was performed. A prolonged T_1_ relaxation time of ∼1751 ms was detected. The native myocardium has a T_1_ relaxation time of ∼1240 ms and the left ventricular blood of 1774 ms, which is similar to that of the tumour, suggesting a potential vascular nature of the structure (*Figure 2*). T_1_ mapping showed isointensity to the myocardium, and T_2_ mapping showed a homogenous hyperintense mass with heterogenous late enhancement. No further enlargement was detected.

**Figure 3:**
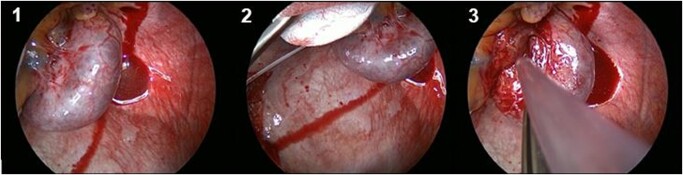
Intraoperative view of an epicardial cavernous haemangioma (*) in the region of the inferiorvena cava, right atrium, and coronary sinus before surgical excision from three different angles.

An abdominal sonography showed several hypoechoic lesions in the liver segments VII (2 × 2 cm), V (1.9 × 1.3 cm), and IV (1.7 × 1.6 cm) without requiring surgical intervention. No further investigations were performed, as we had to prioritize an intervention for the growing epicardial mass. Echocardiographic control revealed an interatrial position of the tumour suggesting compression of both atria. The compression signs and the necessity to clarify the nature of the tumour strongly indicated surgical intervention. A full sternotomy approach with the support of cardiopulmonary bypass (CPB) via bicaval cannulation of the superior and inferior vena cava was performed. The ascending aorta (AAo) was cross-clamped, followed by administering an antegrade Calafiore cardioplegic solution. The lowest intraoperative temperature was 34.5°C. A complete excision of the tumour in one segment was successfully performed and was sent for histopathology. No invasion of adherent structures was seen; thus, no reconstruction was necessary, and no margins were taken (*Figure 3*). The total aortic clamping time was 50 min, while CPB and total operation time were 74 and 154 min, respectively.

Histopathology confirmed a cavernous haemangioma (5 × 4.5 × 2 cm) without signs of malignancy. A CD31 stain was seen to be positive. The Ki67 marker MIB1 showed a minimal proliferation activity of 1–2%.

The patient was readmitted due to a postoperative arrhythmia. After medication adjustments, no further complications arose. At 1 year follow-up, there are no signs of recurrence, and the patient reports a restored physical resilience, no dyspnoea, or episodes of palpitations.

## Discussion

Haemangiomas are rarely observed in the cardiac region.^3^ This benign tumour was found near the vena cava and right atrium. This location has previously been described in one quarter of all documented cases.^3^ The patient was experiencing symptoms of palpitations and dyspnoea upon exertion. A TTE was performed, which first revealed an epicardial hyperechoic mass. A differential diagnosis of this tumour can be made by location and appearance. Multimodality imaging is further necessary to investigate vascularization and the nature of the structure. A contrast-enhanced CT angiography was performed. The differential diagnosis included a paraganglionic mass, as it is often found in atrial walls. While most paragangliomas are benign, there is a possibility of malignancy.^6^ The most important differential diagnosis was a malignant angiosarcoma, often located near the right atrium with extensive invasion of adjacent structures. Cardiac CT gives insights into local invasion, while MRI reveals vascularization and haemodynamic effects of the tumour.^12^ A definite diagnosis can be confirmed by histopathology. Coronary angiography was performed to assess any feeding arteries. A partial connection to the RCA could not be ruled out. During resection, no connection to the coronary vessels was observed.

In the absence of obstructive symptoms, physicians suggest observing and monitoring the cardiac mass and its progression via CT or MRI.^1^ Another approach used in infantile cardiac haemangiomas is medical therapy via propranolol,^13^ which has not been employed in adult patients. Clear guidelines for the management of cardiac haemangiomas are currently lacking.^1^ In this case, compression signs were detected, but the benign nature was unclear, as structures were found in the abdominal region and a partial connection to the RCA could not be ruled out. Therefore, surgery was indicated. In the recent years, a totally endoscopic resection on-pump beating heart (TES ON-BH) approach for the removal of cardiac masses has emerged, as it has shown advantages such as lower infection rates and less blood loss.^14^ However, due to the assumption that the RCA might be feeding the tumour, the surgeon had to be prepared for a more extensive intervention. The optimal access was via full sternotomy on total CPB.

Surprisingly, the tumour had no connection to the distal RCA. A complete surgical excision was performed. Histopathology confirmed a benign cavernous haemangioma.

## Conclusion

Cardiac cavernous haemangiomas are inherently benign and rarely undergo malignancy. The necessity of surgical intervention remains controversial, as most patients are asymptomatic, and their discovery is often incidental. Surgical excision or sampling stands as the definitive intervention and diagnostic approach. Our knowledge about cardiac haemangiomas is primarily drawn from case reports, leaving a lack of clear guideline-based treatments. Many case reports,^1,10,15^ including the one we are presenting, demonstrate favourable outcomes following surgical intervention.

Performing multimodality imaging to differentiate cardiac masses is imperative. Although cavernous tumours exhibit slow growth, they might be supplied by coronary vessels or induce compression on cardiac structures, necessitating differentiation from aggressive malignant angiosarcomas. Risk assessment is crucial, given the potential for life-threatening complications such as tamponade or even sudden death.

## Supplementary Material

ytae146_Supplementary_Data

## Data Availability

No new data were generated or analysed in support of this case report.

## Lead author biography

Rayan Cheaban, MD, is a resident in thoracic cardiovascular surgery at the Heart and Diabetes Center in Nord Rhine Westphalia, which is one of the leading hospitals for heart and lung diseases as well as all forms of diabetes. Each year, we successfully treat patients with the most complex disease entities, having the chance to treat critically ill patients in various disciplines.

## Supplementary material

**Consent:** The patient signed an informed written consent for submission and publication of this case report including images and associated text according to the journal’s patient policy in line with COPE guidance.

**Funding:** None declared.
